# Is leptin receptor expression triggered in the case of embryo transfer to endometrium coculture?

**DOI:** 10.3906/sag-1810-160

**Published:** 2019-08-08

**Authors:** İskender KAPLANOĞLU, Gülnur TAKE KAPLANOĞLU, Özgür ÇINAR, Güleser GÖKTAŞ, Serdar DİLBAZ, Cemile Merve SEYMEN

**Affiliations:** 1 Center of Assisted Reproduction, Etlik Zübeyde Hanım Women’s Health Teaching and Research Hospital, Sağlık Bilimleri University Ankara Turkey; 2 Department of Histology and Embryology, Faculty of Medicine, Gazi University, Ankara Turkey; 3 Department of Histology and Embryology, Faculty of Medicine, Ankara University, Ankara Turkey; 4 Department of Histology and Embryology, Faculty of Medicine, Lokman Hekim University, Ankara Turkey

**Keywords:** Endometrium, embryo culture, embryo, leptin

## Abstract

**Background/aim:**

A synchronized dialogue between maternal and embryonic tissues is required for successful implantation. Low uterine receptivity is responsible for two-thirds of implantation failures and leptin is effective in the physiology of reproduction by binding to specific receptors. In this study, we investigate leptin receptor expression in cases of embryo transfer to endometrial coculture.

**Materials and methods:**

Biopsy materials were taken from 20 females with indication for coculture application and were cultured in an appropriate medium after the epithelial cells were isolated. The grown cells were cultured in chamber slides as the first group. For the second group, day 3 embryo was added to chamber slides and the development was observed. The embryo was transferred 1 or 2 days later and other cells (after the transfer process) were used to form the second group. After fixation, immunohistochemical staining was performed with anti-leptin primary antibody.

**Results:**

Regarding the coculture without embryo transfer, moderate leptin receptor immunoreactivity was seen in the perinuclear region and the cell membrane. Also, regarding the coculture with embryo transfer, moderate leptin receptor immunoreactivity was seen in the cytoplasm and strong leptin receptor immunoreactivity was seen in the cell membrane.

**Conclusion:**

Embryo transfer to endometrium coculture triggers leptin receptor expression.

## 1. Introduction

Infertility is an important public health problem. Around 40%–50% of cases are due to female infertility, whereas 30% of cases are linked to male infertility. There is no identifiable causative factor defined in 15%–25% of the couples [1]. Reproductive biotechnologies such as artificial insemination (AI), embryo transfer (ET), and embryo production via in vitro fertilization (IVF) have improved significantly in recent years [2]. However, the rate of live births per embryo transfer through assisted reproductive technologies remains as low as 30%, so there is still a substantial need to improve current human IVF-ET procedures by determining optimal embryonic culture conditions [3]. For patients with repeated IVF failures, the methods to improve IVF outcomes include focusing on improvement in embryo quality and implantation rates, because repeated IVF failure occurs due to unsuccessful implantation, according to the generally accepted view [4]. 

A receptive endometrium, a normal and functional embryo at the blastocyst developmental stage, and a synchronized dialogue between maternal and embryonic tissues are required for successful implantation [5]. Among these requirements is the window of implantation, which is the interaction between an embryo and a receptive endometrium during a well-defined period in the secretory phase of the menstrual cycle [6]. 

This embryo-maternal crosstalk is mediated by a variety of biochemical factors and cytokines produced by the endometrium and the blastocyst [6,7]. Progesterone (P) receptors, estrogen receptors, leukemia inhibitory factor, transforming growth factor beta, and tumor necrosis factor alpha are among these factors. Integrins, mucins, and cytokines such as interleukin 15 and leptin also play essential roles as adhesion molecules [8–10].

Several techniques have been used to improve culturing conditions to closely mimic these important interactions in the in vivo environment in IVF. Embryo coculture systems using helper cells are one of such strategies. They have been applied for patients with a history of poor embryo quality or with repeated implantation failures. The target of embryo coculture systems is to improve embryonic development during the critical period of maternal-to-zygotic transition and to increase implantation potential [11,12]. The release of embryotrophic factors and the detoxification of the culture medium by removal of heavy metal cations, free radicals, or metabolic inhibitors are some of the mechanisms targeted by coculture [12,13].

Based on this information, in this study our aim is to investigate****the leptin receptor expression in cases of embryo transfer to endometrium coculture.

## 2. Materials and methods

### 2.1. Obtaining endometrial coculture cells 

In accordance with routine IVF procedures, endometrial materials of approximately 0.5 cm3 were obtained via vacuum aspiration from 20 patients with indication for coculture application. These patients did not have PCOS syndrome. The materials were placed into Falcon tubes that included sterile u291b modified Eagle’s medium (DMEM). They were kept in an oven for a period of about 1 h, during which time the suspension was shaken for 5–10 min and was then centrifuged at 1600 rpm for 10 min. Afterwards, the supernatant was removed, and the medium was put on the pellet, which was then followed by resuspending. The tissue suspension was planted into a 100-mL dish. The next day, the holding cells were obtained and the top medium as well as the tissue debris were removed, after which fresh medium was added. The daily development of the cells was observed. The bottom of the dish was covered by cells within 3–4 weeks, and the cells were removed from the dish by trypsin. This cell suspension was centrifuged, and the supernatant was removed. Fresh medium was added to the pellet and thus a new cell suspension was obtained. The new suspension was planted into 5 different central well dishes. It was expected that the cells would proliferate and fill the wells in 3 to 7 days. If the IVF cycle started in a short time, the suspension was used immediately. A day 3 embryo was put into the first central well dish. The dishes were changed every day, and day 5 embryo transfer was performed. 

After the culturing process, the grown cells were cultured in chamber slides as in the first group. For the second group, a day 3 embryo was added to the chamber slides and the development was observed, which was followed by transfer 1 or 2 days later. The other cells (after the transfer process) were used to form the second group. The first group of cells was kept in the same environment under equal conditions for the same period of time. 

### 2.2. Immunohistochemical study

After creating the groups, the slides were fixed in 4% paraformaldehyde for about 30 min. Then the slides were washed twice in phosphate-buffered saline (PBS) solution for 15 min. The endogenous peroxidase activity was blocked with 3% hydrogen peroxide (Fisher Scientific, Melrose Park, IL, USA) (diluted with PBS) for 15 min and the slides were washed three times in PBS solution. The epitopes were stabilized by applying serum blocking solution for 15 min. The slides were incubated with the anti-leptin receptor primary antibody for 60 min at room temperature. The slides were washed three times in PBS solution, after which the biotinylated secondary antibody was applied for 10 min. The slides were then washed three times in PBS solution. Thereafter, streptavidin peroxidase was applied to the slides for 10 min. After washing with PBS, 3,3’-diaminobenzidine (DAB) was used as a chromogen. Then all the slides were counterstained with Harris’s hematoxylin. The slides were examined with a photo-light microscope (DM4000B Image Analysis System, Leica, Germany) and a Leica DFC280 Plus camera. 

### 2.3. Statistical study

The number of immunopositive cells was measured manually using the Qwin software program in consecutive areas. The following semiquantitative scoring system was used to assess the immunolabeling intensity: (0) no staining, (1) weak, (2) weak to moderate, (3) moderate, (4) moderate to strong, and (5) strong labeling. Two independent observers who were blind to the treatment protocol performed the evaluations of immunolabeling scores independently. The H-score was calculated using the following equation: H-score = ∑Pi (i + 1), where i is the intensity of Leptin-receptor labeling with a value of 0, 1, 2, 3, 4, or 5, and Pi is the percentage of labeled cells for each intensity, varying from 0% to 100%. The results are expressed as mean ± SD.

Statistical analysis was performed using unpaired Student t-tests for the H-score, using SigmaStat v3.5 software. The results are expressed as mean values ± SD, and P < 0.05 was considered statistically significant.

## 3. Results

**Figure 1:**
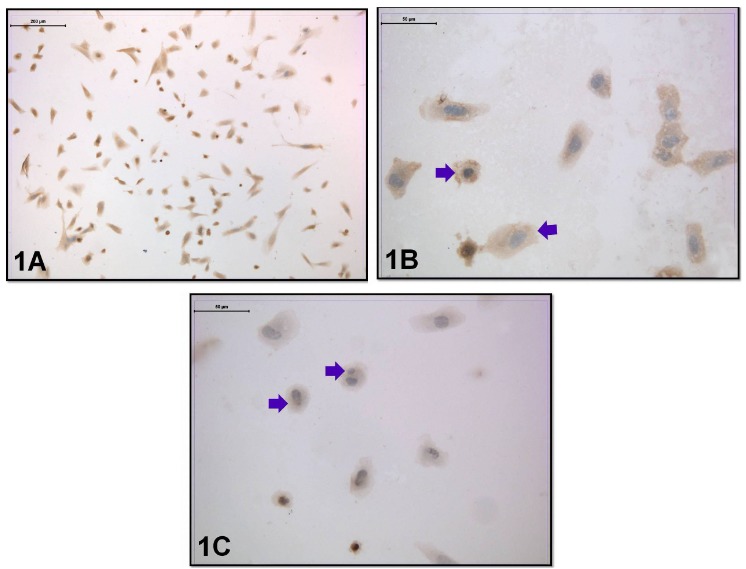
Leptin-receptor immunoreactivity was seen in the coculture without embryo transfer. Arrow: Moderate immunoreactivity in the perinuclear region and in the cell membrane (immunoperoxidase-hematoxylin; A 100×; B, C 400×).

Cells were obtained with centrally located nuclei and normal cytoplasmic appearance in the coculture without embryo transfer. In a group of cells, a double nuclear formation was observed due to amitotic division. The distribution of the leptin receptor was evaluated in this group. Moderate immunoreactivity was seen in the perinuclear region and the cell membrane (Figures 1A–1C).

**Figure 2:**
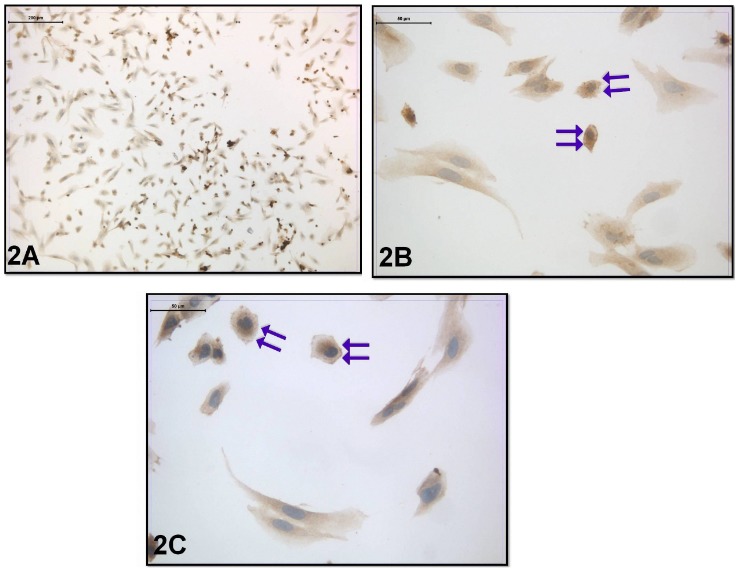
Leptin-receptor immunoreactivity was seen in the coculture with embryo transfer. Double Arrow: Moderate cytoplasmic and strong membranous immunoreactivity (immunoperoxidase-hematoxylin; A 100×; B, C 400×).

Regarding the coculture with embryo transfer, the cell morphology was not significantly different from the previous group. However, when the distribution of leptin receptor was evaluated in this group, moderate leptin receptor immunoreactivity was seen in the cytoplasm. Strong leptin receptor immunoreactivity was also seen in the cell membrane (Figures 2A–2C).

**Figure 3:**
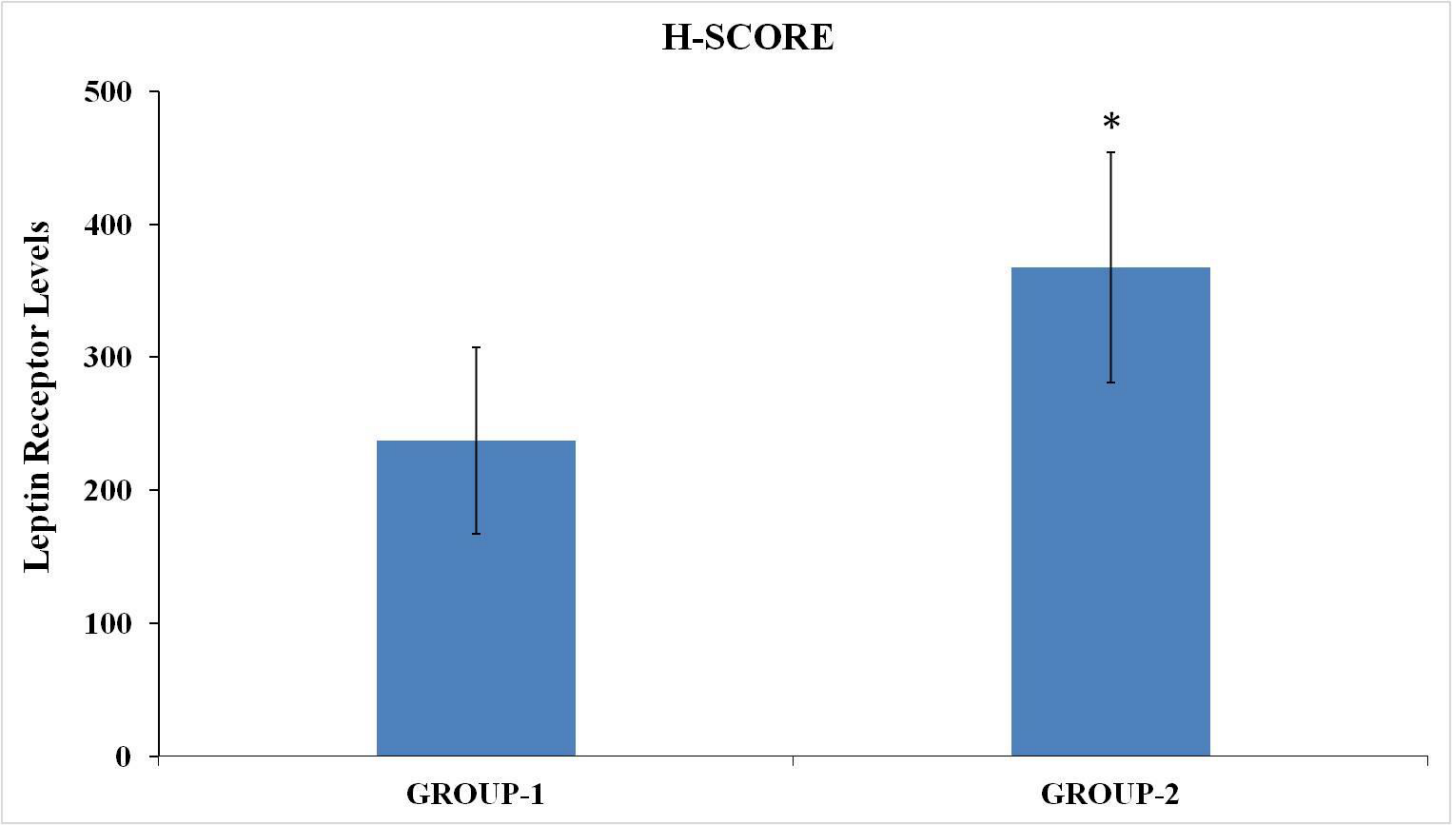
Statistical diagram of leptin-receptor levels as a result of H-score analyses.

These findings were also supported by statistical analysis (Figure 3). The data were expressed as mean ± SD for each group. Statistical significance was determined with unpaired Student t-tests, *P < 0.05, versus Group 1.

## 4. Discussion

Leptin, a 16-kDa product of the Obese (*Ob*) gene, is primarily produced by adipose tissue. The leptin system (leptin and its receptors) plays a role in food intake, basal metabolism and reproductive functions/fertility [14]. Indeed, leptin is an autocrine and paracrine regulator of the human implantation process [15]. For example, according to several studies, leptin causes synergistic stimulation of angiogenesis and vascular permeability via FGF-2 and VEGF also increases the expression of MMP-2 and 9. Angiogenesis and metalloproteinases are essential for the success of implantation, so leptin and leptin receptors have been detected in implantation sites and this detection is very important for preimplantation and implantation physiology [16].

Several cytokines like leptin and adiponectin are localized at the maternal/fetal interface, and they are significant modulators of human embryo implantation and placentation [17].

According to some studies, leptin-deficient mice cannot reproduce [18,19], and leptin promotes mouse preimplantation embryo development [20]. Moreover, in a study by Yang et al., leptin was found to enhance the adhesion and outgrowth of mouse blastocysts on fibronectin [21]. Some of these findings were also supported by studies in which human blastocysts were cocultured with endometrial epithelial cells due to modulation of leptin secretion [15].

At present, little is known about leptin and adiponectin system regulation in the human endometrium. We found strong immunoreactivity, especially for the cell membrane in embryo-transferred coculture, so our results showed that embryo transfer to endometrium coculture triggered leptin receptor expression.

According to a study, the deficiency of leptin synthesis is related to obesity and sterility in ob/ob knock-out mice [22]. Moreover, another study suggested that higher leptin concentrations have been secreted by competent blastocysts than in the case of arrested embryos. Gonzales et al. also found that leptin and leptin receptor mRNA and protein were revealed in secretory endometrium and in endometrial epithelial cells that were cocultured with human embryos [15]. 

Taken together, the results of this study suggest that day 3 embryo transfer to endometrium coculture triggers leptin receptor expression as one modulator of human embryo implantation.
